# Sr/PTA Metal Organic Framework as A Drug Delivery System for Osteoarthritis Treatment

**DOI:** 10.1038/s41598-019-54147-5

**Published:** 2019-11-26

**Authors:** Zhen Li, Ying Peng, Xingyu Xia, Zhe Cao, Yuqing Deng, Bin Tang

**Affiliations:** 1 Department of Biomedical Engineering, Southern University of Science and Technology, Shenzhen, 518055 P.R. China; 2 Department of Physics and Key Laboratory of Artificial Micro- and Nano-structures of Ministry of Education, School of Physics and Technology, Wuhan University, Wuhan, 430072 P.R. China; 3 Department of Mechanical Engineering, University of Hong Kong, Hong Kong, China; 4 Guangdong Provincial Key Laboratory of Cell Microenvironment and Disease Research, Guangdong, P.R. China; 5 Shenzhen Key Laboratory of Cell Microenvironment, Shenzhen, 518055 P.R. China

**Keywords:** Biomaterials, Diseases

## Abstract

A Sr-based metal-organic framework (MOF) is introduced as ketoprofen carrier to form a comprehensive system for treating osteoarthritis (OA), and the drug loading amount and release rate is investigated. Structural characterization of the samples showed that Sr/PTA-MOF had good crystal morphology and structure, and chemical and thermal stability. Ketoprofen was successfully loaded on the MOF carrier, which had been identified by high performance liquid chromatography (HPLC) and thermogravimetric analysis (TGA). The release experiment manifested that more than 90% of ketoprofen released from Sr/PTA-MOF after 24 h, and ketoprofen delivery was mainly governed by the Higuchi model. Furthermore, cytotoxicity experiment manifested that synthesized MOF carrier had no poisonous effect on OA chondrocytes, which provided a preliminary foundation for the realization of comprehensive treating OA.

## Introduction

Osteoarthritis (OA) is known as a primary cause of disability and functional incapacity in adults worldwide^1^ which is largely associated with episodic pain, physical disability, and a reduction in quality of life. Recent evidence indicates an association of osteoarthritis with increased risk of mortality, and the prevalence of osteoarthritis rises markedly from the age of 50 years^2^. Inflammation, pain and unbalanced subchondral bone are three key factors, which lead to the progressive deterioration of OA. Firstly, inflammatory factors can up-regulated the release of various matrix proteases and inhibit collagen formation to degrade extracellular matrix. Secondly, pain can lead to decreased mobility in OA patients, which causes the loss of bone in the subchondral bone and severe cases even develop osteoporosis. Finally, the unbalanced subchondral bone can increase the friction of the cartilage. The three factors may affect one another, feeding the violent cycle^3–5^. Therefore, these three key factors must be addressed if effective treatment of OA is to be achieved.

Ketoprofen, 2-(3-benzoylphenyl)- propionic acid, a member of the non-steroidal anti-inflammatory drugs (NSAIDs), has potent analgesic and antipyretic properties, making it effective in the long-term management of OA, rheumatoid arthritis, ankylosing spondylitis, *et al*.^6^. It a good choice to be used for eliminating inflammation and analgesia. On the other hand, strontium (Sr) is a trace element in the human body, which has become increasingly popular in the prevention and treatment of osteoporosis as a ranelate compound^7^. Recently, clinical studies reported SrRan to be of potential interest for OA patients. For example, Bruyere *et al*. reported that SrRan can reduce the progression of radiographic features of spinal OA and back pain in women with osteoporosis and concomitant spinal OA^8^. Besides, Tat *et al*. thought that strontium ranelate may exert a positive effect on OA pathophysiology by inhibiting the synthesis of key factors leading to bone resorption, a feature associated with the OA process^7^. In addition, many works proved that Sr also had the effects of reducing the inflammatory processes and relieving pain, which can be a good auxiliary ketoprofen effect.

In recent years, drug delivery strategies have been developed, which can increase the residence time of drugs molecule so that can enhance the efficacy in treating OA. Various of different materials are acted as drug carriers to control release of the drug dosage, such as polymers, alginate, lipids, chitosan, *et al*. However, results have been unsatisfactory due to the low loading amount or poorly controlled release with an absence of tunable porosity^9^. Currently, metal organic frameworks (MOFs), composed of metals (or metal clusters) and organic linkers, have gained attention in this field, and can be a promising candidates as drug carriers because of their remarkably large surface areas and excessively high porosities^10^. Besides, a high structural flexibility can adapt their pores to accommodate the shape and size of organic molecules, making it advantageous over rigid nanoparticle carriers in biomedical applications^11^. Researchers have made much effort to introduce MOFs for drug delivery systems as drug carriers in recent years. For example, Nadizadeh *et al*. synthesized Cu-MOFs and used for controlled ibuprofen drug delivery, and exhibited well-defined drug release behavior^12^. Férey and co-workers synthesized two flexible MOFs, MIL-53(Cr) and MIL-53(Fe), and showed that both MOFs could carry ~20 wt% of ibuprofen and complete delivery of ibuprofen was achieved under physiological conditions after 3 weeks^13^. In our previous study, we have proved that Zr-based MOFs were successfully loading and releasing ketoprofen^14^.

Therefore, in this paper, we will utilize the functions of ketoprofen and Sr to build a comprehensive treatment system for OA. We will prepare Sr-based MOF as a drug carrier of ketoprofen, then discuss the physical and chemical properties of synthesized MOF, and study the ketoprofen molecule loading amount and drug release properties. Meanwhile, the effect of cytotoxicity was also investigated.

## Results

### Characterization of synthetic MOFs

**Figure 1:**
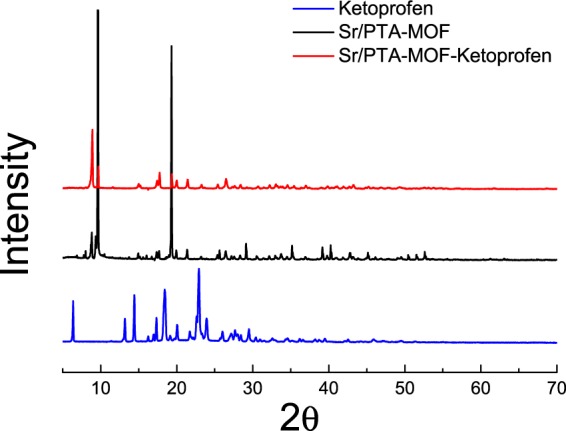
XRD analysis of Sr/PTA-MOF before and after loading ketoprofen.

Figure 1 shows the XRD patterns of Sr/PTA-MOF before and after loading ketoprofen. The XRD pattern of synthesized Sr/PTA-MOF presented good crystal structure and was well coincided with that in literature report^15^, manifesting that the MOF carrier was successfully synthesized. When loading ketoprofen, all of the diffraction peaks are not shift, exhibiting no structural changes. However, some modifications in the intensities ratio of Bragg reflections can be seen probably due to some interatomic distances and bond angles was changed or the preferential orientation of crystallites imposed during drug loading process^16^, or due to the thin gelatinous film on the surface of Sr/PTA-MOF referring to the results of SEM^17^. Besides, characteristic peaks of ketoprofen were hard to find, which might be most of ketoprofen was encapsulated inside the MOF carrier or the low content. Furthermore, the crystal structure of MOF carrier was little affected, indicting the synthesized MOF carrier were stable.

**Figure 2:**
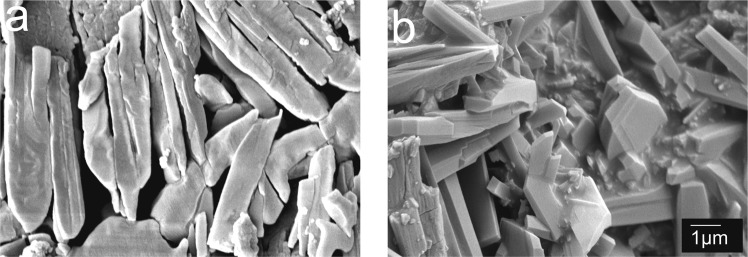
The scanning electron microscopy (SEM) of Sr-based MOFs before and after loading ketoprofen: (**a**) Sr/PTA-MOF, (**b**) Sr/PTA-MOF-Ketoprofen.

To obtain microscopic morphological and structural information, scanning electron microscopy (SEM) analyses of Sr-based MOFs are performed, as shown in Fig. 2. Sr/PTA-MOF presented rod-like, indicated that prepared it had good crystal morphology. After loading drug, it can be seen a slight gelatinous film attached on the surface of Sr/PTA-MOF, which might be ketoprofen adsorbed on the carrier.

The nitrogen adsorption-desorption isotherms are in Fig. S1. The N2 adsorption-desorption isotherm of all adsorbents were assigned to type IV according to the IUPAC classification and exhibited a hysteresis at high relative pressure, indicated the structure of carrier was not damaged.

**Figure 3:**
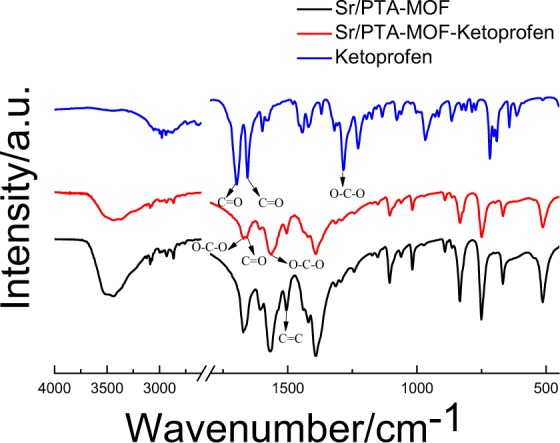
The FTIR analysis of Sr/PTA-MOF before and after loading ketoprofen.

Figure 3 shows the Fourier transform infrared (FTIR) spectra of the synthesized samples and loading drug MOFs. For ketoprofen, the peak at around 1697 cm^−1^ was due to C=O stretching of carboxylic group^18^, which was not found in MOF carriers, indicated that there was no H_2_BDC ligand residue during the synthesis process of Sr-based MOF. Besides, the peak at approximately 1655 cm^−1^ is attributed to C=O stretching of ketonic group^18^.

For Sr/PTA-MOF, the peak at around 1685 cm^−1^ and 1395 cm^−1^ are assigned to -O-C-O in the carboxylate group of the BDC ligand^19^, and the peak located at about 1575 cm^−1^ was attributed to the possible reaction of -COOH with Sr^20^, Furthermore, the energy band at around 1506 cm^−1^ was attributed to the typical vibration of the C=C in the benzene ring^21^. After loading ketoprofen, the characteristic peaks of Sr/PTA-MOF-Ketoprofen were not changed, indicated the good chemical stability. Besides, the C=O stretching of ketonic group at approximately 1655 cm^−1^ was manifested on the surface of Sr/PTA-MOF-Ketoprofen, indicating the ketoprofen was successfully loaded on the drug carrier.

### Drug loading of Sr-based MOFs

The loading amount of ketoprofen on drug carriers are tested by high performance liquid chromatography (HPLC). And the absorption maximum is determined by UV-Vis spectra analysis.

The UV-Vis spectra of ketoprofen in NaOH solution is shown in Fig. S2(a). The absorption maximum was at 256 nm, which was then used in HPLC test. Figure S2(b) shows the relation between the concentration of ketoprofen and the peak areas of HPLC test, showing a very good correlation coefficient >0.99.

Table S1 shows the loading amount of ketoprofen of synthesized Sr-based MOF. Sr/PTA-MOF presented 36% loading, indicated it had a higher ketoprofen loading amount.

**Figure 4:**
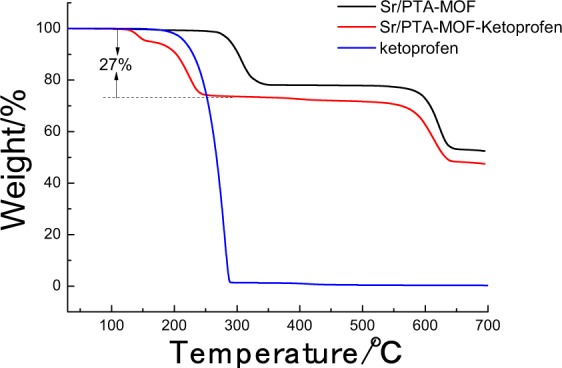
The thermogravimetric (TG) analysis of Sr/PTA-MOF before and after loading ketoprofen.

The thermogravimetric (TG) analysis is performed to realize the thermal stability of MOFs and assess the amount of ketoprofen, shown in Fig. 4. Sr/PTA-MOF was thermal stable up to 265 °C, and there was a weight loss of about 22% between 265 and 350 °C, which was corresponded to the removal of the coordinated DMF molecule, and the second weight loss procedure began around 500 °C, corresponding to the framework collapse of MOFs. After loading ketoprofen, the weight loss of Sr/PTA-MOF began at around 125 °C, which might be the decomposition of ketoprofen referred the TG analysis of ketoprofen, and the ketoprofen amount was about 27% based on the weight loss. However, the was no weight loss at about 265 °C, which might be the DMF molecules was removed during the loading process of ketoprofen.

### Release of ketoprofen

The release of ketoprofen was carried out *in vitro* in phosphate buffer saline (PBS). UV-vis spectra profile of ketoprofen in PBS solution is shown in Fig. S3(a), and the maximum wavelength is observed at 260 nm. Figure S3(b) shows the relation between the concentration and absorbance of ketoprofen, indicated a very good correlation coefficient.

**Figure 5:**
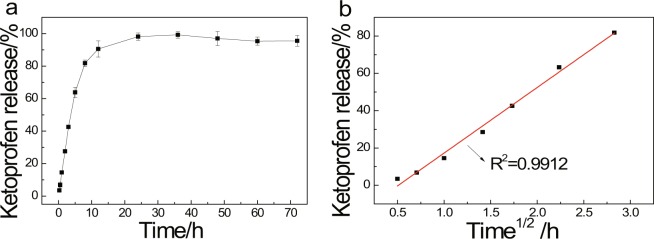
(**a**) Ketoprofen release from Sr/PTA-MOF in PBS; (**b**) Fitting of the ketoprofen delivery data to a Higuchi model.

The ketoprofen release behavior of the Sr-based MOF composite was investigated by using UV–vis spectroscopy, shown in Fig. 5(a). There are two stages for ketoprofen release: a quick release at the beginning 8 h, and slow release after 8 h. Combination with the previously published literature, the releasing process can be divided into three steps: when ketoprofen molecules was far away from the wall of pore, the intermolecular interaction on the surface of MOFs was the main cause, and a burst release happened; when ketoprofen molecules existed close to the walls of cages, the interactions between the acid group of drug molecule and the carboxylic groups of the porous material, such as hydrogen bonds would affect the ketoprofen release, and the release rate reduced; For those ketoprofen encapsulated in these pores, ketoprofen was be released in a slow rate under the influence of pore adsorption, hydrogen bonds and intermolecular interactions, *et al*. The amount of ketoprofen released from Sr-based MOF was approximately 80% after 8 h, and then gradually reached above 90% after 24 h, which indicated the good controlled release of ketoprofen. The quick release at the beginning 8 h might be due to the ketoprofen molecules release, existed on the surface and close to the cage walls of MOFs.

To study the release kinetics and the involved mechanism, the first 8 hours of ketoprofen delivery were fitted to three mathematical model: zero-order, first order and Higuchi model^18,20^. Table 1 shows the coefficient values (R^2^) of various model. It was manifested that the Higuchi model was the most suitable for describing the releasing rate in Sr-based MOF, shown in Fig. 5(b). And ketoprofen delivery can be explained by Eq. (1):

1 $$[{\rm{drug}}]=K\cdot {t}^{1/2}$$

where [drug] corresponds to the concentration of released drug (mg · g^−1^), t is the time (h), and K is the kinetic constant (g · mg^−1^ · h^−1/2^). This indicates that the drug release is here mainly governed by a diffusion process, predictable by the Higuchi model and dependent on several factors such as the structure (dimensionality, interconnectivity, pore size) and composition (polarity, interactions).

**Table 1:** Correlation coefficient (R^2^) obtained for each mode.

Mathematical model	Correlation coefficient (R^2^)
	Sr/PTA-MOF
Zero-order	0.9699
First-order	0.7557
Higuchi model	0.9912

### *In vitro* cytotoxicity

**Figure 6:**
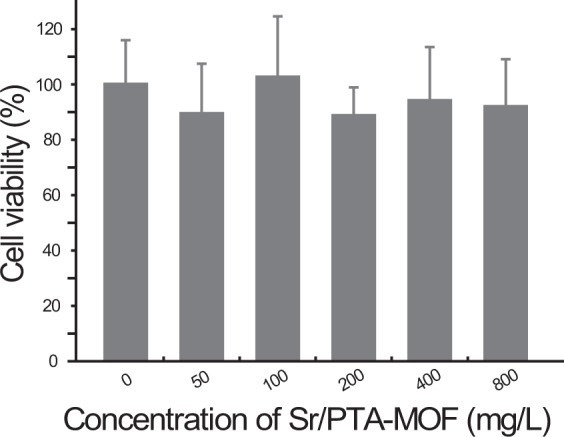
MTT cytotoxicity assay of Sr/PTA-MOF towards chondrocyte at various concentrations.

Given Sr/PTA-MOF has the high ketoprofen loading amount and slow release rate, the vitro cell viability of Sr/PTA-MOF was evaluated by the reduction activity of the methyl thiazolyl tetrazolium (MTT, [3-(4,5-dimethylthiazol-2-yl)-2,5- diphenyltetrazolium bromide]) assay to study the bio-toxicity of Sr/PTA-MOF. Figure 6 shows the viability results of chondrocyte incubated with different concentrations of Sr/PTA-MOF.

There was no significant change in the proliferation of chondrocyte for 24 h at 37 °C incubator for different concentrations of MOFs. the cellular viability was still greater than 90% when incubated at a concentration up to 800 mg · L^−1^. Furthermore, the differences in means were analyzed and indicated there was no significant difference (t-test, *p* > 0.05) between the experimental groups and the control group. manifesting that the synthesized Sr/PTA-MOF was a good biocompatible material without toxicity and was be suitable as the delivery vehicle for drugs.

## Discussion

**Figure 7:**
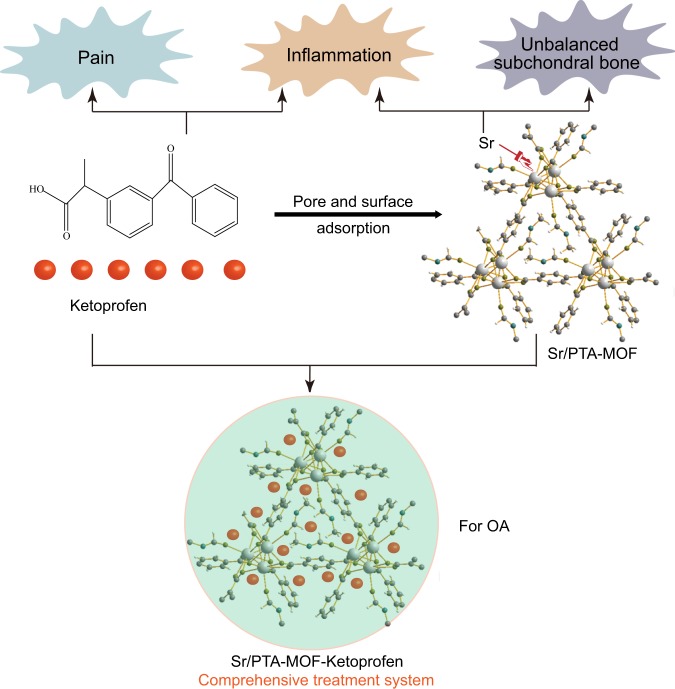
Sr/PTA-MOF-Ketoprofen was considered as comprehensive treatment system of OA.

In summary, Sr/PTA-MOF was synthesized successfully, presented good crystal structure and stability. Ketoprofen was successfully loaded on the MOF carrier having little effect on the crystal structure of MOFs. The loading amount of ketoprofen of Sr/PTA-MOF was 36%. Most ketoprofen released after 24 h due to existing on the surface and pore of Sr/PTA-MOF. Ketoprofen delivery was mainly governed by the Higuchi model dependent on several factors such as the structure (dimensionality, interconnectivity, pore size) and composition (polarity, interactions). It ensures that ketoprofen can achieve anti-inflammatory and analgesic effects when Sr/PTA-MOF-Ketoprofen is first introduced into the body. Furthermore, cytotoxicity experiment manifested that synthesized Sr/PTA-MOF had no poisonous effect on OA chondrocytes. Therefore, Sr/PTA-MOF-Ketoprofen could be considered as a comprehensive treatment system of OA, which was a promising compound for anti-inflammation, analgesia and maintaining bone balance (Fig. 7).

## Methods

### Materials

All chemicals used for Sr-based MOF preparation were of analytical grade. Strontium nitrate nonahydrate (Sr(NO_3_)_2_, >99%) and N,N′-dimethylformamide (DMF, 99.5%) were purchased from Aladdin Co. (Shanghai, China); Glacial acetic acid (CH_3_COOH, >99.5%) were purchased from Shanghai Ling Feng Chemical Reagent Co., Ltd. (Shanghai, China).

### Preparation of MOFs

The syntheses of strontium terephthalic acid MOF (Sr/PTA-MOF) was carried out following these steps^15^. SrCl_2_ · 6H_2_O (0.58 g, 2.2 mmol), H_2_BDC (0.32 g, 1.93 mmol) and 56 mL DMF were mixed and stirred, then placed in a Teflon-lined stainless steel vessel at 100 °C for 2 days. After cooled to room temperature, synthesized MOFs was filtered, washed with DMF and dried under vacuum at 60 °C.

### Characterization of Sr/PTA-MOF

Synthesized Sr/PTA-MOF was characterized and analyzed using various techniques. The microstructure of MOFs was analyzed by Field Emission Scanning Electronic Microscope (FESEM) (ZEISS GeminiSEM). The X-ray diffraction (XRD) patterns were obtained by X-ray diffractometer (Bruker D8 Advance ECO, German) using CuKα radiation (λ = 1.5418 Å). A spectrometer (BRUKER VERTEX 70, USA) in KBr plates was used to record the Fourier transform infrared (FT-IR) spectra (4000–400 cm^−1^). The UV-Vis absorption spectra were recorded on a spectrophotometer (PerkinElemer Lambda 750S, USA). The thermal stabilities of the MOFs were assessed using a TGA/DSC1 (TGA 550, USA), and the ramp rate for the thermogravimetric analysis (TGA) was 10 °C min^−1^ from 30 to 700 °C. Furthermore, the loading amount of ketoprofen was measured by high performance liquid chromatography (HPLC) (Agilent 1260 LC, USA). Nitrogen adsorption-desorption isotherms were obtained by using nitrogen-adsorption apparatus (micromeritics ASAP 2020), and all samples were degassed for 8 h at 150 °C.

### Ketoprofen loading

2.5 g of ketoprofen was dissolved in 20 ml of ethyl alcohol at room temperature, and then 150 mg of Sr/PTA-MOF was added in this solution. After stirring for 24 h, the nanoparticles were obtained by filtration and washed with distilled water, and finally dried at 150 °C for 24 h to further treat. The loading samples were labeled as Sr/PTA-MOFs-Ketoprofen.

Loaded ketoprofen amount in MOFs is measured as following: 5 mg of samples after ketoprofen loading were digested separately in 10 mL of NaOH (0.1 mol/L) overnight. Then 2 mL of supernatant was taken and then mixed with 2 mL of methanol. After filtering by 0.45 µm filter membrane, this solution was tested using Agilent 1260 LC high performance liquid chromatography. The mobile phase was V(acetonitrile): V(K_2_HPO_4_, pH = 2) = 1:1. And sunfire-C18 reverse-phase column (5 μm, 4.6 × 150 mm Waters) was employed. Besides, the flow rate was 1 mL · min^−1^ and the column temperature was fixed at 25 °C. Ketoprofen in NaOH solutions (0.05, 0.1, 0.15, 0.2, 0.25, 0.3, 0.35, 0.4, 0.45, 0.5 mg · mL^−1^) were used as standards.

### Drug release

The drug release was studied by dispersing 20 mg of Sr/PTA-MOF into 200 mL of phosphate buffer saline (PBS) containing tween 80 (0.5% v/v) at pH 7.4 and stirred constantly for 72 h. 5 mL of supernatant was taken out to calculate the drug release using by UV-Vis (at 260 nm) at different time point. Meanwhile, 5 mL of freshly preheated PBS solution was added to the mother liquor to keep the equilibrium of total solution.

The amount of ketoprofen loading was determined with high performance liquid chromatography (HPLC) and TGA. And the release percentages of ketoprofen were calculated according to the formula, release percentage (%) = m_r_/m_l_, where m_r_ is the amount of released ketoprofen, while m_l_ is the total amount of loaded ketoprofen.

### *In vitro* cytotoxicity

Chondrocyte was cultured in dulbecco’s modified eagle medium (DMEM) high glucose containing 1% penicillin-streptomycin and 10% fetal bovine serum at 37 °C. The vitro cytotoxicity of Sr/PTA-MOF was evaluated by methyl thiazolyl tetrazolium (MTT) assays against chondrocyte. Briefly, chondrocyte was plated in a 96-well plate with a density of 1 × 10^4^ cells/well, cultured for 24 h. Then, 100 μL of Sr/PTA-MOF with various concentrations were added and incubated for 24 h. 10 μL of MTT (5 mg/mL) were added and incubated for another 4 h after the cell medium was replaced and washed with PBS. Finally, the MTT-formazan generated by live cells was dissolved in 100 μL of dimethyl sulfoxide (DMSO), and the absorbance at 490 nm of each well was monitored using the iMark Microplate Reader. The cytotoxicity was estimated by the relative cell viability (%) compared with the untreated control cells.

## Supplementary information

supporting information
