# Supplementary material for: Sr/PTA Metal Organic Framework as A Drug Delivery System for Osteoarthritis Treatment
Source: Sci Rep. 2019 Nov 26;9:17570. doi: 10.1038/s41598-019-54147-5 (PMC6879484; doi:10.1038/s41598-019-54147-5)
Supplement: Supplementary file 1 — supporting information [file 41598_2019_54147_MOESM1_ESM.pdf]

Sr/PTA Metal Organic Framework as A Drug Delivery System for Osteoarthritis Treatment

Zhen Li[1,2], Ying Peng[1], Xingyu Xia[3], Zhe Cao[1], Yuqi Deng[1], Bin Tang[*1,4,5]

[1]Department of Biomedical Engineering, Southern University of Science and Technology, Shenzhen 518055, P. R. China. Email: tangb@sustc.edu.cn

[2]Department of Physics and Key Laboratory of Artificial Micro- and Nano-structures of Ministry of Education, School of Physics and Technology, Wuhan University, Wuhan 430072, P. R. China.

[3]Department of Mechanical Engineering, University of Hong Kong, Hong Kong, China.

[4]Guangdong Provincial Key Laboratory of Cell Microenvironment and Disease Research, Guangdong P. R. China.

[5]Shenzhen Key Laboratory of Cell Microenvironment, Shenzhen 518055, P. R. China.

Table S1 Ketoprofen payloads in the different MOFs estimated by HPLC

	Sr/PTA-MOF-load (w%)
Content (w%)	36±0.8

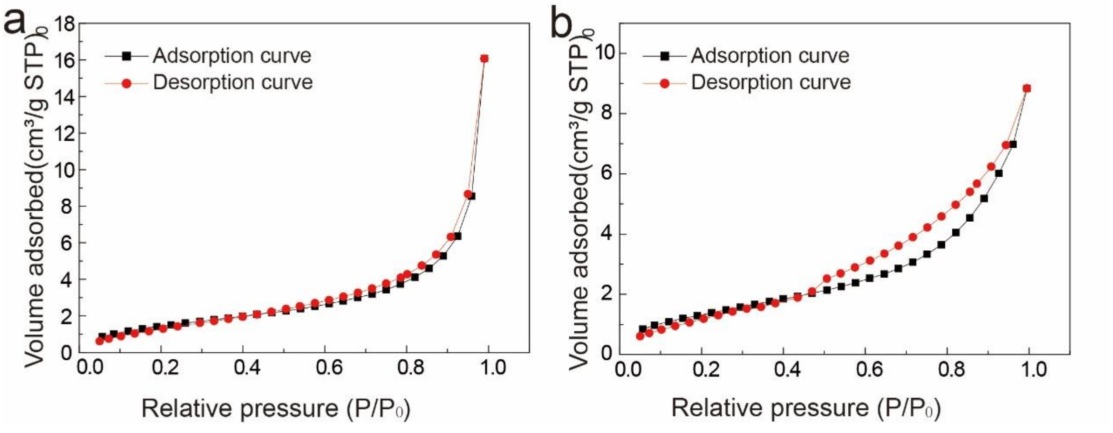

Figure S1. Nitrogen adsorption-desorption isotherms. (a) Sr/PTA-MOF; (b) Sr/PTA-MOF-Ketoprofen.

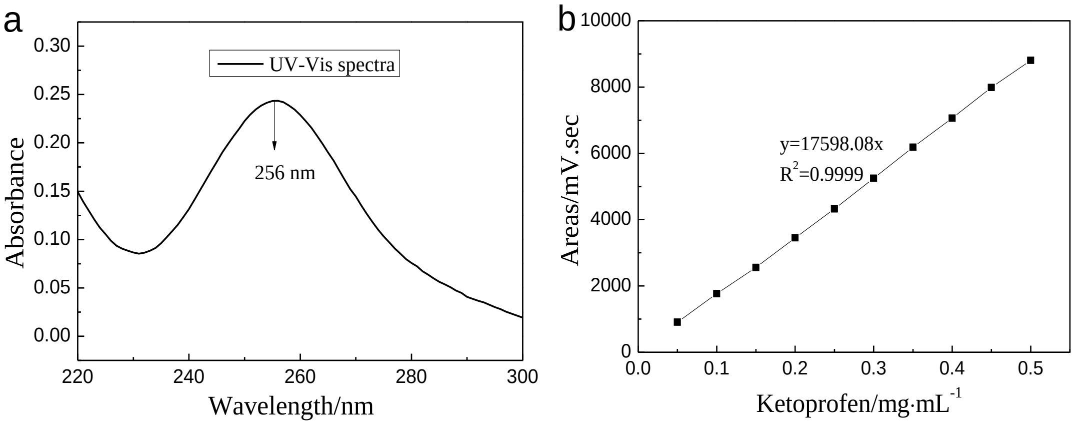

Figure S2. (a) UV-Vis spectra of ketoprofen in NaOH solution; (b) Calibration plot of standard ketoprofen by HPLC method.

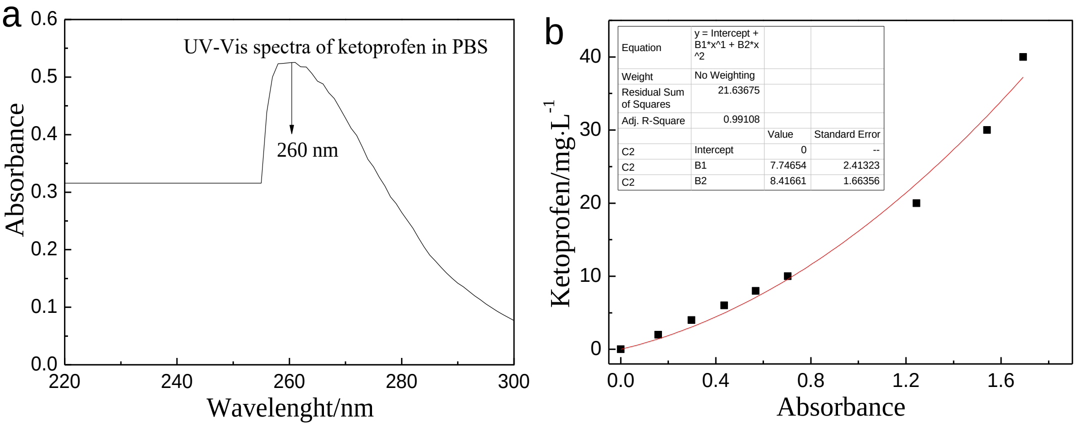

Figure S3. (a) UV-Vis spectra of ketoprofen in PBS solution; (b) Calibration plot of standard ketoprofen by UV-vis spectra
